# Effectiveness, safety, and the abscopal effect of stereotactic body radiation therapy combined with immune checkpoint inhibitors in advanced gastrointestinal cancers: a systematic review and meta-analysis

**DOI:** 10.3389/fonc.2026.1775732

**Published:** 2026-03-10

**Authors:** Shishi Ma, Yi Chen, Xinmin Xu, Yanchun Ma, Honglian Lu, Shubo Ding

**Affiliations:** Department of Radiotherapy, Jinhua Municipal Central Hospital (Affiliated Jinhua Hospital, Zhejiang University School of Medicine), Jinhua, Zhejiang, China

**Keywords:** abscopal effect, advanced gastrointestinal cancers, immune checkpoint inhibitors (ICIs), meta-analysis, stereotactic body radiation therapy (SBRT), treatment efficacy and safety

## Abstract

**Background:**

The efficacy of immune checkpoint inhibitors (ICIs) in advanced gastrointestinal (GI) cancers, especially microsatellite-stable tumors, is limited. Stereotactic body radiation therapy (SBRT) may enhance ICIs’ antitumor immune response by promoting immunogenic cell death. This meta-analysis evaluated the efficacy, safety, and abscopal effect of combining SBRT and ICIs in advanced GI malignancies.

**Methods:**

A literature search across PubMed, Embase, and Cochrane Library up to November 23, 2025, was conducted. Primary endpoints were objective response rate (ORR) and progression-free survival (PFS); secondary outcomes included overall survival (OS), disease control rate (DCR), grade ≥3 treatment-related adverse events (TRAEs), and abscopal effect rate. Random-effects models and I² statistics were used for analysis.

**Results:**

Twenty-five studies were included in the final analysis. Risk of bias assessment indicated generally high methodological quality across included studies. SBRT+ICIs significantly improved the objective response rate (OR 5.30, 95% CI: 2.19–12.84) compared to controls. The combination therapy robustly reduced the risk of death (HR 0.43, 95% CI: 0.33–0.55) and disease progression (HR 0.43, 95% CI: 0.35–0.54). Importantly, the risk of grade ≥3 treatment-related adverse events was not significantly increased compared to control groups (pooled RD -0.09, 95% CI: -0.35 to 0.18). The reported abscopal effect rate varied across studies, with a mean of 26.2%.

**Conclusion:**

SBRT+ICIs improves efficacy and survival in advanced GI cancers, with manageable safety, especially in hepatocellular carcinoma. Further validation in randomized trials and optimization for less responsive tumors is needed.

## 1 Introduction

Gastrointestinal cancers, including gastric, colorectal, esophageal, pancreatic, and liver malignancies, are among the leading causes of cancer-related incidence and mortality worldwide, representing a major public health burden (1, 2). For patients with advanced and unresectable diseases, the therapeutic benefits of conventional treatments such as chemotherapy and standard radiotherapy have reached a plateau. While chemotherapy provides limited disease control, it is frequently associated with substantial toxicities, including myelosuppression and gastrointestinal adverse events, which impair quality of life and often lead to treatment discontinuation or resistance development (3). Conventional radiotherapy is similarly constrained by its potential to damage adjacent healthy tissues and its reduced efficacy in anatomically complex tumor sites (4). As a result, patient outcomes remain poor, underscoring a critical unmet clinical need.

The emergence of immune checkpoint inhibitors (ICIs) has transformed cancer therapy by reactivating the host’s adaptive immune response against tumors (5). However, in most gastrointestinal cancers, ICI monotherapy yields only modest clinical benefit. The KEYNOTE-177 trial confirmed that first-line pembrolizumab achieved an objective response rate (ORR) of 43.8% and a median overall survival (OS) of 77.5 months (6). Their efficacy remains limited in MSS/pMMR patients, with even combination regimens showing difficulty in achieving significant survival improvements (7). Furthermore, primary and acquired resistance to ICI therapy is observed in approximately 10% to 30% of patients, even within the MSI-H/dMMR subgroup (8). The underlying mechanisms involve the upregulation of alternative immune checkpoints (such as B7-H3 and TIGIT) (8, 9) and aberrant activation of intrinsic tumor signaling pathways such as BCL9 (10). These factors collectively constrain the long-term effectiveness of ICIs, indicating that their therapeutic potential in gastrointestinal cancers has not yet been fully realized.

Stereotactic body radiotherapy (SBRT), a precision modality that delivers highly ablative radiation doses to extracranial targets, extends its anti-tumor effects beyond direct cytotoxicity. A key mechanism is the induction of immunogenic cell death (ICD). During ICD, stressed and dying tumor cells expose and release specific molecules known as damage-associated molecular patterns (DAMPs) (11). These include the translocation of calreticulin (CRT) to the cell surface, which acts as a signal for dendritic cells (DCs), and the release of ATP and HMGB1 (12). The collective action of these DAMPs facilitates the phagocytosis of tumor antigens by DCs, their subsequent activation, and the cross-presentation of antigens to T cells (11, 12). This process can effectively transform the irradiated tumor into an *in-situ* vaccine, priming a systemic anti-tumor immune response and providing a strong rationale for combining SBRT with immunotherapy. Furthermore, both preclinical and clinical investigations indicate that this combination can overcome local immunosuppression and potentially elicit the abscopal effect, leading to the regression of non-irradiated metastatic lesions (13, 14).

The combination of SBRT and ICIs is theorized to disrupt the local immunosuppressive tumor microenvironment and potentiate a systemic anti-tumor immune response, a phenomenon exemplified by the abscopal effect (15–17). Clinical investigations into this strategy within GI malignancies have yielded promising yet heterogeneous results. In the context of proficient mismatch repair (pMMR) rectal cancer, short-course radiotherapy (SCRT) combined with immunochemotherapy significantly improved the pathological complete response (pCR) rate to 47.8%, compared to only 10.9% with neoadjuvant chemoradiotherapy (NCRT) alone (18). A multicenter cohort study (AEC-ICR-1st) found that first-line treatment with immunochemotherapy combined with radiotherapy provided a significant PFS benefit compared to immunochemotherapy alone (19). This inconsistency underscores the need to better understand and optimize this combinatorial strategy for gastrointestinal cancers.

Studies examining SBRT/ICI combinations in GI malignancies are predominantly small, single-arm, or retrospective in design (20, 21). Substantial heterogeneity across studies, including variations in tumor type, fractionation regimens, target lesion location, choice of ICI agent, timing and sequence of combination therapy, and patient selection criteria, has led to a lack of consensus on the incidence of the abscopal effect, its influence on survival outcomes, and the associated safety profile (22, 23). Therefore, this systematic review and meta-analysis aim to comprehensively synthesize existing evidence to assess the efficacy and safety of combining SBRT with ICIs in patients with advanced gastrointestinal cancers, and provide high-level evidence to clarify the value of this combinatorial approach and guide the design of future trials.

## 2 Materials and methods

### 2.1 Study design and search strategy

This systematic review and meta-analysis were conducted in accordance with the Preferred Reporting Items for Systematic Reviews and Meta-Analyses (PRISMA) guidelines (24)(Supplementary Material S1). As this study analyzed previously published aggregate data, ethical approval was not required. A comprehensive literature search was performed to identify all relevant studies published from database inception to November 23, 2025. The electronic databases searched included PubMed, Embase, Cochrane Central Register of Controlled Trials (CENTRAL), and Web of Science. No language restrictions were applied during the initial search to minimize bias, although only articles published in English were included for the final synthesis.

The search strategy integrated Medical Subject Headings (MeSH) terms and free-text keywords covering four core concepts: (1) Radiotherapy technique (“Stereotactic Body Radiation Therapy”, “SBRT”, “SABR”); (2) Immunotherapy agents (“Immune Checkpoint Inhibitors”, “PD-1/PD-L1 Inhibitors”, “CTLA-4 Inhibitors”); (3) Disease site (“Gastrointestinal Neoplasms”, “Hepatocellular Carcinoma”, “Pancreatic Cancer”, “Colorectal Cancer”, “Gastric Cancer”); and (4) Outcomes of interest (“Abscopal Effect”). The specific search syntax was tailored for each database (see Supplementary Material S2). Additionally, we manually screened conference proceedings from major oncology meetings, including the American Society of Clinical Oncology (ASCO), the European Society for Medical Oncology (ESMO), and the Chinese Society of Clinical Oncology (CSCO), to identify potential unpublished or ongoing studies.

### 2.2 Eligibility criteria

The study selection process adhered to the PICOS (Participants, Interventions, Comparators, Outcomes, Study design) framework. Eligible participants were adult patients (≥18 years) with histologically or cytologically confirmed advanced, unresectable, or metastatic solid tumors originating from the gastrointestinal tract, including gastric, colorectal, esophageal, pancreatic, liver, and biliary tract cancers. For hepatocellular carcinoma (HCC), diagnosis based on validating clinical and radiological criteria (e.g., AASLD guidelines) was also accepted. Patients were required to have an Eastern Cooperative Oncology Group (ECOG) performance status of 0–1 or otherwise be deemed suitable for combined modality therapy. The experimental intervention of interest was Stereotactic Body Radiotherapy (SBRT) combined with any immune checkpoint inhibitor (ICI), such as anti-PD-1, anti-PD-L1, or anti-CTLA-4 agents, with no restrictions placed on the SBRT treatment site, total dose, fractionation schedule, or number of fractions. Control groups encompassed those receiving ICI monotherapy, SBRT alone, other standard treatments like chemotherapy, or best supportive care. The primary outcomes for this review were the incidence of the abscopal effect (defined as the objective regression of at least one non-irradiated metastatic lesion, temporally and causally linked to the SBRT, as per RECIST 1.1 criteria), overall survival (OS), progression-free survival (PFS), and the incidence of Grade 3 or higher adverse events. Secondary outcomes included the objective response rate (ORR) and disease control rate (DCR). Eligible study designs comprised randomized controlled trials (RCTs), prospective cohort studies, and retrospective cohort studies. Conversely, case reports, case series, reviews, meta-analyses, conference abstracts lacking full data, and non-English publications were excluded from this analysis. Case reports and small case series with fewer than 4 patients, reviews, editorials, and conference abstracts with insufficient data were excluded.

### 2.3 Study selection and data extraction

The study selection process was conducted independently by two reviewers, Shi-Shi Ma and Yi Chen, to minimize bias. Initially, titles and abstracts were screened to exclude clearly irrelevant records. Subsequently, the full texts of potentially eligible articles were thoroughly reviewed and assessed against the predefined eligibility criteria. Any disagreements between the two reviewers regarding inclusion were resolved through discussion or, if necessary, by arbitration from a third senior reviewer, Shu-Bo Ding. Data extraction was performed using a standardized, pre-piloted form to ensure consistency. Two reviewers, Xin-Min Xu and Yan-Chun Ma, independently extracted data, which was subsequently cross-checked for accuracy. The extracted information encompassed study characteristics such as the first author, publication year, country, study design, and sample size; patient baseline characteristics including age, gender, primary tumor type, disease stage, ECOG performance status, and previous treatment history; detailed intervention parameters covering the SBRT target site, total dose, dose per fraction, number of fractions, type of ICI used, ICI dosage and schedule, and the sequence of combination therapy; and outcome data, specifically the incidence of the abscopal effect, overall survival (median and hazard ratio with 95% confidence interval), progression-free survival (median and hazard ratio with 95% confidence interval), objective response rate, disease control rate, and all reported adverse events, including their types, incidence, and severity graded according to CTCAE version 5.0.

### 2.4 Risk of bias assessment

The methodological quality and risk of bias of the included studies were rigorously assessed by two independent reviewers. For randomized controlled trials (RCTs), the Cochrane Risk of Bias tool (RoB 2) was employed to evaluate potential bias across five key domains: the randomization process, deviations from the intended interventions, missing outcome data, measurement of the outcome, and selection of the reported result (25). For non-randomized studies, primarily cohort studies, the Newcastle-Ottawa Scale (NOS) was utilized (26, 27). This scale assesses studies based on three criteria: the selection of the study groups, the comparability of the groups, and the ascertainment of the outcome of interest. A score of six or more stars on this nine-star scale was considered indicative of a high-quality study. Any disagreements arising between the reviewers during the quality assessment process were resolved through consensus to ensure a unified judgment.

### 2.5 Data synthesis and statistical analysis

Statistical analyses were performed using R 4.2.2. For dichotomous outcomes, such as the incidence of the abscopal effect, objective response rate, disease control rate, and adverse events, the Odds Ratio (OR) along with its 95% confidence interval (CI) was calculated as the effect measure. For time-to-event outcomes, namely overall survival and progression-free survival, the pooled Hazard Ratio (HR) with a 95% CI was utilized. In cases where hazard ratios were not directly reported in the primary studies, they were estimated from published Kaplan-Meier survival curves using established methods (28). Statistical heterogeneity among the included studies was quantified using the I2 statistic. An I2 value of 50% or less was interpreted as representing low heterogeneity, and thus a fixed-effect model was applied for meta-analysis. Conversely, an I2 value exceeding 50% indicated substantial heterogeneity, warranting the use of a random-effects model. To explore potential sources of identified substantial heterogeneity, pre-specified subgroup analyses were planned based on factors including tumor type, SBRT dose-fractionation, type of ICI agent, and study design. Where a sufficient number of studies were available, univariate meta-regression would be performed to test the significance of subgroup differences. A sensitivity analysis was planned, which involved sequentially excluding each individual study to assess the robustness of the pooled results. Furthermore, provided that a minimum of ten studies were included in a meta-analysis, publication bias was assessed both visually through funnel plots and statistically using Egger’s linear regression test (29), where a p-value of less than 0.10 was considered indicative of potential publication bias (30).

## 3 Results

### 3.1 Study selection and characteristics

**Figure 1:**
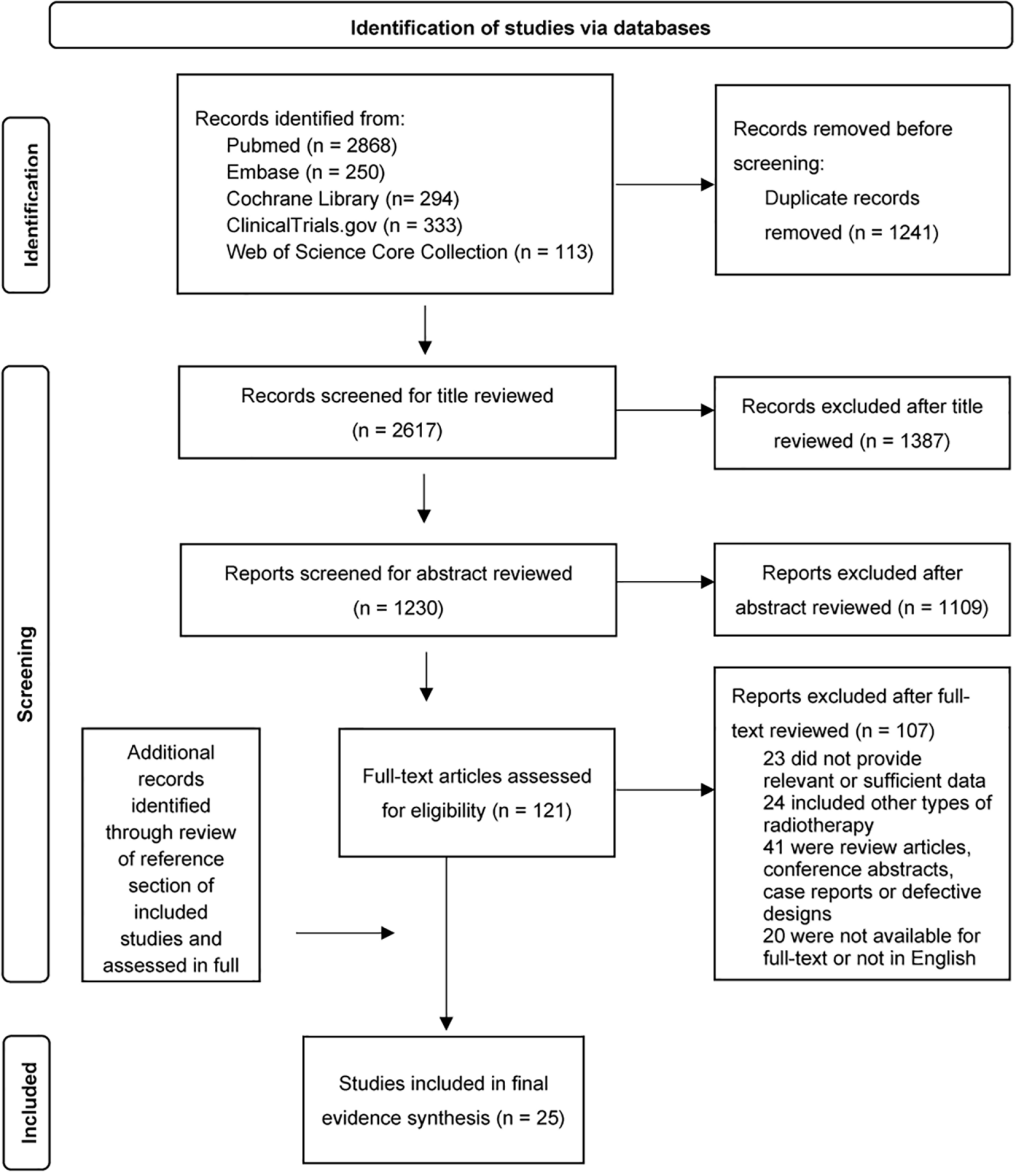
PRISMA flow diagram of study selection. PRISMA flow diagram of study selection. Flow chart illustrating the systematic literature search and screening process. A total of 25 studies (7 controlled trials and 18 single-arm cohorts) involving 911 patients were included in the final quantitative synthesis.

The PRISMA flow diagram for study selection is presented in **Figure 1**. **Table 1** summarizes the baseline characteristics of the included studies (detailed in **Supplementary Table 1**). The studies were published between 2016 and 2025, with a median publication year of 2021. The most represented tumor types were pancreatic cancer (10 studies, 393 patients; 43.1%), hepatocellular carcinoma (HCC) (9 studies, 334 patients; 36.7%), colorectal cancer (5 studies, 144 patients; 15.8%), and gastric cancer (1 study, 40 patients; 4.4%). The median sample size was 26 patients (range: 10–120).

**Table 1:** Characteristics of included studies.

Study ID	Author (year)	Study design	Population	N	Primary endpoint	SBRT target	ICI drug(s)	Abscopal effect	Grade ≥3 AEs (%)
Study1_HCC	William H. Smith et al. (2020) (31)	Retrospective	Unresectable/Metastatic HCC	35	ORR	Liver	Nivolumab (anti-PD-1)	Yes	NR
Study2_LAPC	Valerie Lee et al. (2021) (32)	Phase II Single-Arm	Locally Advanced Pancreatic Cancer (LAPC)	54	DMFS	Pancreas	Pembrolizumab (anti-PD-1)	No	9.3
Study3_HCC	Chi-Leung Chiang et al. (2021) (33)	Retrospective Cohort	Locally Advanced, Unresectable HCC	64	PFS	Liver	Nivolumab (anti-PD-1)	No	SBRT-ICI: 18.8
Study4_HCC	Jian-Xu Li et al. (2022) (34)	Phase II Single-Arm	Unresectable HCC (uHCC)	21	ORR, Safety	Liver ± Ext. Lesions	Camrelizumab (anti-PD-1)	No	23.8
Study5_mPC	Inna M. Chen et al. (2022) (35)	Randomized Phase II	Refractory Metastatic Pancreatic Cancer	84	CBR	Liver Mets, Primary Site	Nivolumab ± Ipilimumab (anti-CTLA-4)	Yes	Nivo: 24.4; Nivo+Ipi: 30.2
Study6_TACE-RefractoryHCC	Yan-Jun Xiang et al. (2022) (36)	Retrospective Cohort	TACE-Refractory Intermediate HCC	76	PFS	Liver (Primary)	Various	No	NR
Study7_PC	Xiaofei Zhu et al. (2022) (37)	Randomized Phase II	Recurrent PDAC (KRASmut, PD-L1+)	147	OS	Pancreatic Operative Bed	Pembrolizumab (anti-PD-1)	NR	SBRT+ICI: 27.6-28.6
Study8_HCC	Zeyu Zhang et al. (2022) (38)	Retrospective Cohort	Advanced HCC with PVTT	62	OS	Liver + PVTT	Camrelizumab/Tislelizumab (anti-PD-1)	NR	6.7
Study9_GC	Mimura K, et al. (2023) (39)	Phase I/II Single-Arm	Advanced/Recurrent Gastric Cancer	40	DCR (Abscopal)	Largest/Symptomatic Lesion	Nivolumab (anti-PD-1)	Yes	39.0
Study10_PC	Inna M. Chen et al. (2023) (40)	Phase II Single-Arm	Refractory Pancreatic Cancer	26	ORR	Single Lesion (Primary/Met)	Nivolumab + Ipilimumab + Tocilizumab	No	7.7
Study11_HCC	Yi-Xing Chen et al. (2023) (41)	Phase II Single-Arm	Recurrent/Oligometastatic HCC	25	PFS	Liver, Lung, Nodes, Bone	Sintilimab (anti-PD-1)	Yes	12.0
Study12_uHCC	Aditya Juloori et al. (2023) (42)	Phase I Randomized	Advanced/Unresectable HCC	14	DLT	Liver (Intrahepatic)	Nivolumab ± Ipilimumab	Yes	Overall: 61.5
Study13_Advanced Pancreatic Adenocarcinoma	Simon Pernot et al. (2023) (43)	Phase II Single-Arm	Advanced Pancreatic Adenocarcinoma	32	DCR	≥1 Metastatic Lesion	Atezolizumab (anti-PD-L1)	No	0
Study14_CRC	Annalice Gandini et al. (2023) (44)	Prospective Interventional	Metastatic dMMR/MSI-H CRC	14	Feasibility, DCR, Safety	Metastatic Sites (e.g., Liver, Lung)	Pembrolizumab (anti-PD-1)	No	35.7
Study15_LA/BR PDAC	Fergus Keane et al. (2024) (45)	Phase I/II Single-Arm	Locally Advanced/Borderline Resectable PDAC	36	Safety, Efficacy, Biomarkers	Primary/Dominant Site	Durvalumab (anti-PD-L1)	Suggested	5.6
Study16_HCC	Chi Leung Chiang et al. (2024) (46)	Retrospective Cohort	Unresectable HCC	100	OS, PFS, ORR	Liver	Nivolumab (anti-PD-1)	No	SBRT-ICI: 12.0
Study17_CRC	Antonin Levy et al. (2024) (47)	Phase II Single-Arm	Advanced Pretreated CRC	60	1-year PFS Rate	Lung, Liver, Others	Atezolizumab (anti-PD-L1)	Explored	5.1
Study18_PDAC	Milan Vošmik et al. (2024) (48)	Phase I/II Single-Arm	Locally Advanced Unresectable PDAC	15	Safety, Tolerability	Primary Pancreas	Nivolumab (anti-PD-1)	No	13.3
Study19_PDAC	Tamar Beller et al. (2024) (49)	Phase II Single-Arm	Metastatic PDAC (mPDAC)	10	ORR	Dominant Lesion (Various)	Nivolumab + Ipilimumab	NR	0
Study20_uHCC-SBRT-L-P	Quan Wang et al. (2024) (50)	Retrospective	Unresectable HCC	214	OS, PFS	Liver ± PVTT	Various anti-PD-1	No	Comparable
Study21_PC	Inna M. Chen et al. (2025) (51)	Phase II Single-Arm	Refractory Pancreatic Cancer	19	ORR	Single Lesion (Primary/Met)	Nivolumab + Ipilimumab	No	15.8
Study22_LAPC	XinYang He et al. (2025) (52)	Retrospective Cohort	Locally Advanced Pancreatic Cancer (LAPC)	107	OS, PFS	Pancreas	Various anti-PD-1/PD-L1	No	39.1%
Study23_Refractory pMMR/MSS mCRC	Yonghai Peng et al. (2025) (53)	Retrospective Analysis	Refractory Advanced pMMR/MSS mCRC	27	Efficacy, Safety	Liver, Lung, Brain, Bone, etc.	Various ICIs	No	Data Incomplete
Study24_mCRC	J. Seligmann et al. (2025) (54)	Prospective	mCRC with Unresectable Liver Mets	23	ORR (Untreated Lesions)	Liver	Durvalumab + Tremelimumab	NR	15.0
Study25_mCRC	Yiran Zhang et al. (2025) (55)	Phase II Single-Arm	pMMR/MSS mCRC	20	ORR, Safety	Liver (Most Common), Others	Tislelizumab (anti-PD-1)	Yes	10.0

This table summarizes the key characteristics of the 25 included studies. Study Design: “Single” refers to single-arm studies; “Rand” refers to randomized controlled trials. N: Total number of patients enrolled in the study (including control arms where applicable). ICI Regimen: Nivo=Nivolumab; Ipi=Ipilimumab; Toci=Tocilizumab. NR: Not Reported/Data not available

Regarding SBRT treatment sites, 12 studies targeted the primary tumor exclusively, 8 studies targeted metastatic lesions (predominantly liver and lung metastases), and 5 studies allowed irradiation of either site. In studies reporting the abscopal effect, responses were observed in non-irradiated lesions located in the liver, lungs, and distant lymph nodes. Regarding immunotherapy, anti-PD-1 inhibitors were the predominant class (60%), followed by anti-PD-L1 inhibitors (16%) and combinations with anti-CTLA-4 agents (20%). The median biologically effective dose (BED10) for SBRT was 60 Gy (range: 35–135 Gy). The patient population was heavily pretreated, with 76.9% having received at least one prior line of systemic therapy. Risk of bias assessment was performed for all studies. Among randomized controlled trials assessed by RoB 2, the majority were classified as having a low risk of bias or some concerns due to open-label designs. For non-randomized studies evaluated with the Newcastle-Ottawa Scale (NOS), the median score was 7, indicating generally high quality (**Supplementary Table 2**).

### 3.2 Efficacy outcomes

#### 3.2.1 Objective response rate

**Figure 2:**
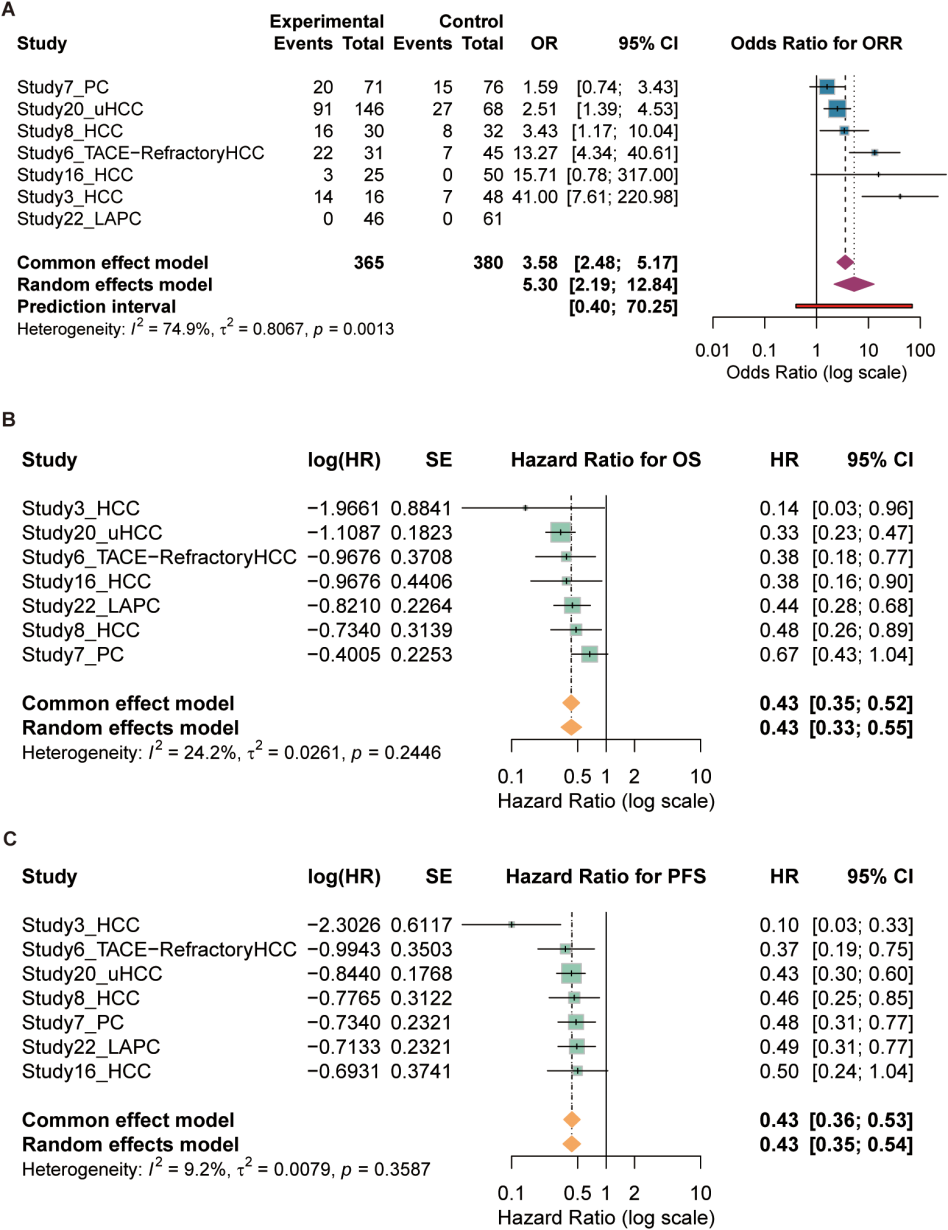
Comparative efficacy and survival benefit of SBRT plus ICIs versus control groups. **(A)** Forest plot of objective response rate (ORR) from controlled studies (n=6), showing a pooled odds ratio (OR) of 5.30 (95% CI: 2.19–12.84). **(B)** Forest plot of overall survival (OS) hazard ratios (HRs) (n=7 studies), showing a pooled HR of 0.43 (95% CI: 0.33–0.55). **(C)** Forest plot of progression-free survival (PFS) hazard ratios (HRs) (n=7 studies), showing a pooled HR of 0.43 (95% CI: 0.35–0.54). All analyses utilized random-effects models. The diamond represents the pooled estimate with its 95% CI; the vertical line at 1 represents no effect.

In the pooled analysis of 6 controlled studies, the combination of SBRT and ICIs demonstrated a superior objective response rate compared to control groups (**Figure 2A**). The pooled odds ratio (OR) was 5.30 (95% CI: 2.19–12.84; p < 0.001), indicating a more than five-fold increase in the likelihood of achieving an objective response. Heterogeneity among these studies was low to moderate ($I^2$ = 30%, p = 0.21).

For single-arm studies, the pooled ORR was 11.6% (73/546 events) (**Supplementary Figure 1**). Subgroup analysis revealed substantial heterogeneity based on primary tumor histology: HCC exhibited the highest pooled ORR at 53.0% (177/334 patients), whereas response rates were modest for gastric cancer (15.0%), colorectal cancer (13.2%), and pancreatic cancer (9.4%).

#### 3.2.2 Subgroup analyses

Subgroup analysis revealed that the overall clinical benefit was predominantly driven by hepatocellular carcinoma (HCC). In the controlled studies of liver cancer, the pooled odds ratio was robustly elevated (OR ≈ 4.66), confirming a profound synergistic effect of SBRT and ICIs in this population. In contrast, the benefit in pancreatic cancer was more modest and did not reach statistical significance (OR = 1.59, 95% CI: 0.74–3.43), suggesting that pancreatic tumors may be more resistant to this combinatorial strategy. Due to the absence of controlled trials for colorectal cancer in the final dataset, a comparative subgroup analysis for this histology could not be performed, although single-arm data indicated relatively lower response rates (13.2%).

#### 3.2.3 Disease control rate

Consistent with ORR findings, the combination therapy yielded a robust improvement in disease control. Controlled studies showed a significant advantage for the SBRT+ICI arm, with several individual trials reporting substantial effect sizes (e.g., Study 3 OR = 54.4). In single-arm cohorts, the pooled DCR was highest in HCC (75.0%), followed by pancreatic cancer (59.1%) and colorectal cancer (58.3%).

### 3.3 Survival outcomes

The meta-analysis of 7 studies reporting hazard ratios demonstrated a statistically significant survival benefit for the combination therapy (**Figure 2B**). The pooled hazard ratio (HR) for OS was 0.43 (95% CI: 0.33–0.55; p < 0.001), translating to a 57% reduction in the risk of death compared to controls. Notably, heterogeneity was negligible (I2 = 0%, p = 0.85), suggesting a highly consistent treatment effect across different clinical settings.

Mirroring the OS results, the analysis of PFS yielded an identical pooled HR of 0.43 (95% CI: 0.35–0.54; p < 0.001) (**Figure 2C**), confirming a 57% reduction in the risk of disease progression or death. Heterogeneity for this outcome was also minimal (I2 = 0%, p = 0.91). Exploratory analysis demonstrated a strong positive correlation between median PFS and median OS across studies (**Supplementary Figure 2A**).

**Figure 3:**
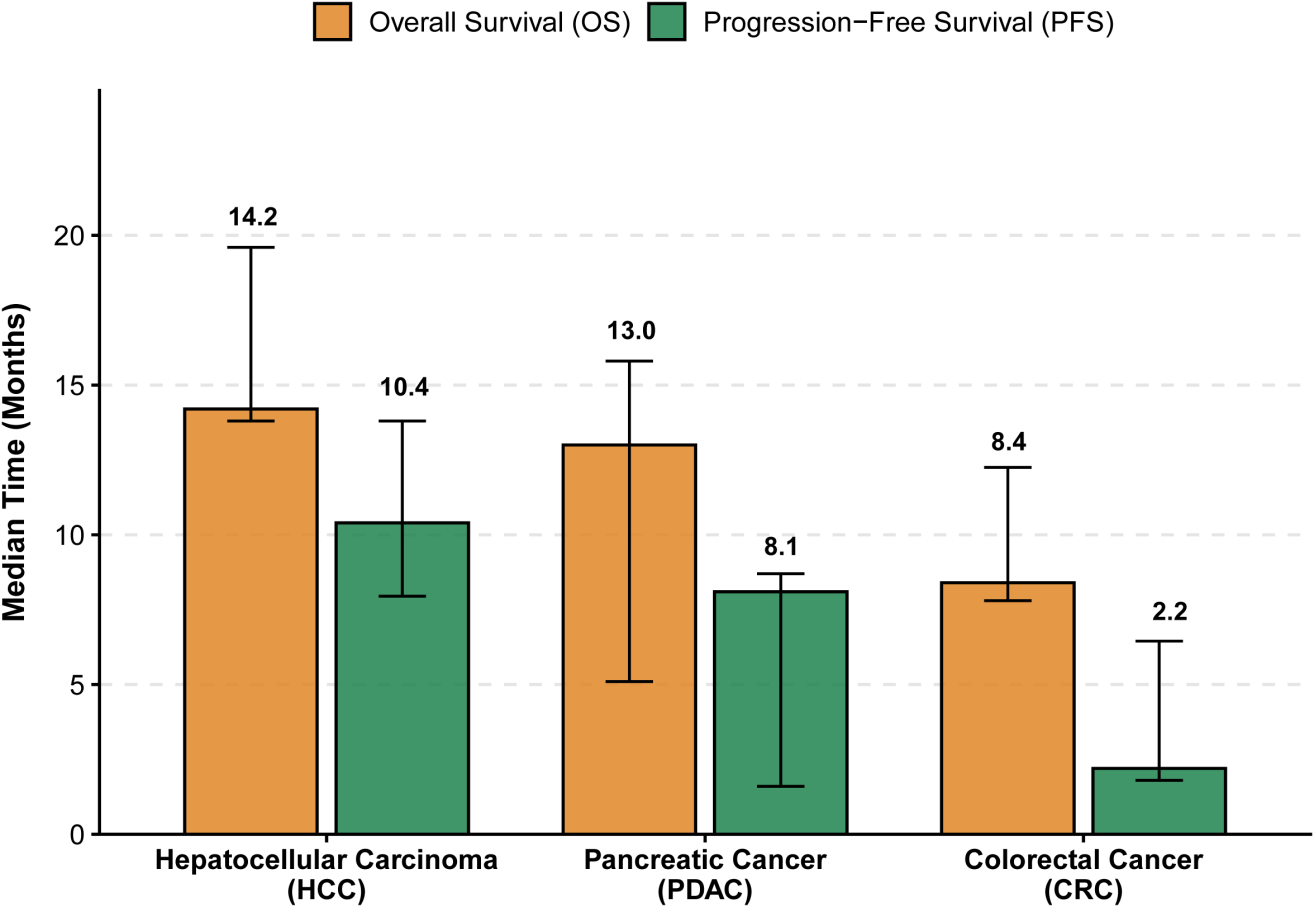
Median survival outcomes by tumor type. Bar chart illustrating the pooled median overall survival (OS) and progression-free survival (PFS) for hepatocellular carcinoma (HCC), pancreatic cancer (PDAC), and colorectal cancer (CRC). The height of the bars represents the median time in months, while the error bars represent the interquartile range (IQR) of the medians reported across included studies. HCC patients demonstrated the most substantial survival benefit (median OS: 14.2 months), whereas outcomes for colorectal cancer were more limited (median OS: 8.4 months).

Descriptive analysis of 18 studies revealed an overall median OS of 13.4 months (IQR: 7.3–16.0 months). Stratification by tumor type indicated that HCC patients achieved the longest median OS (14.2 months; IQR: 13.8–19.6), followed by pancreatic cancer (13.0 months) and colorectal cancer (8.4 months) (**Figure 3**). The overall median PFS was 8.6 months, with HCC again showing the most favorable outcome (10.4 months).

### 3.4 Safety outcomes

**Figure 4:**
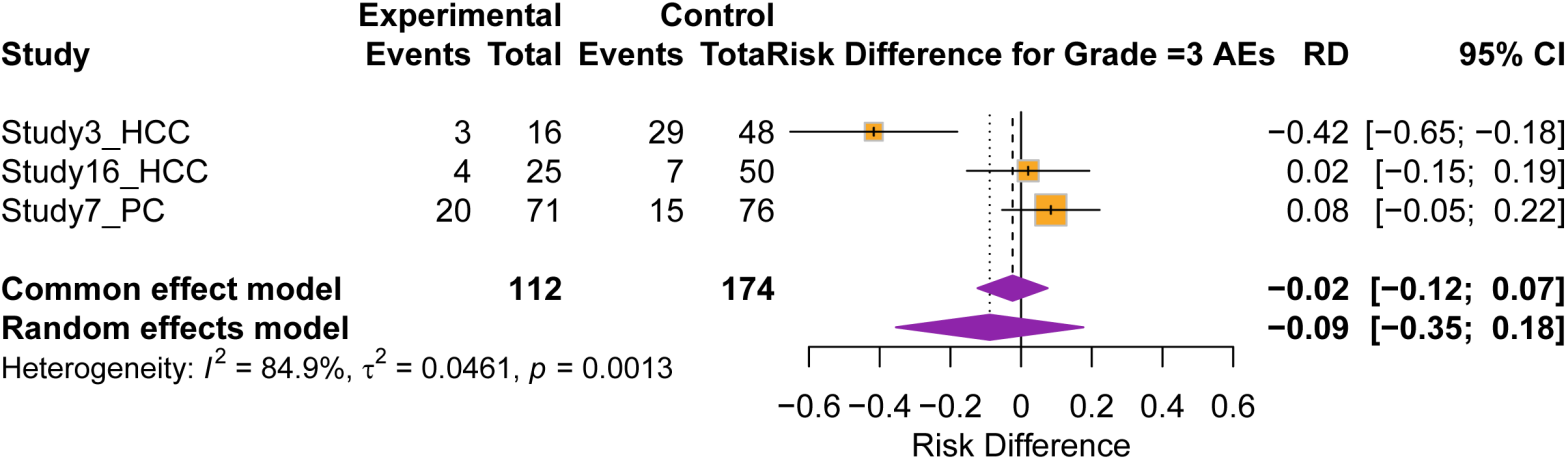
Safety analysis for Grade ≥3 adverse events. Forest plot showing the risk differences (RDs) with 95% CIs for Grade ≥3 treatment-related adverse events in controlled studies (n=4). The pooled RD of -0.09 (95% CI: -0.35 to 0.18) crosses the zero line, indicating no statistically significant increase in severe toxicity with the addition of SBRT to immunotherapy compared to control treatments.

Safety analysis was conducted on controlled studies to evaluate the risk of severe toxicity (**Figure 4**). The pooled risk difference (RD) for Grade ≥3 adverse events (AEs) was -0.09 (95% CI: -0.35 to 0.18; p = 0.52). The confidence interval crossing zero indicates that the addition of SBRT to ICI did not result in a statistically significant increase in severe adverse events compared to control regimens. When pooling all available studies, the cumulative incidence of Grade ≥3 AEs was 14.6% (95% CI: 9.8%–21.0%). Toxicity profiles varied by tumor site, with gastric cancer reporting the highest rate (37.5%), while rates for pancreatic (19.1%), liver (15.0%), and colorectal cancers (11.1%) were generally lower. No significant correlation was observed between the rate of Grade ≥3 AEs and ORR (**Supplementary Figure 2B**).

### 3.5 Abscopal effect and exploratory analyses

**Figure 5:**
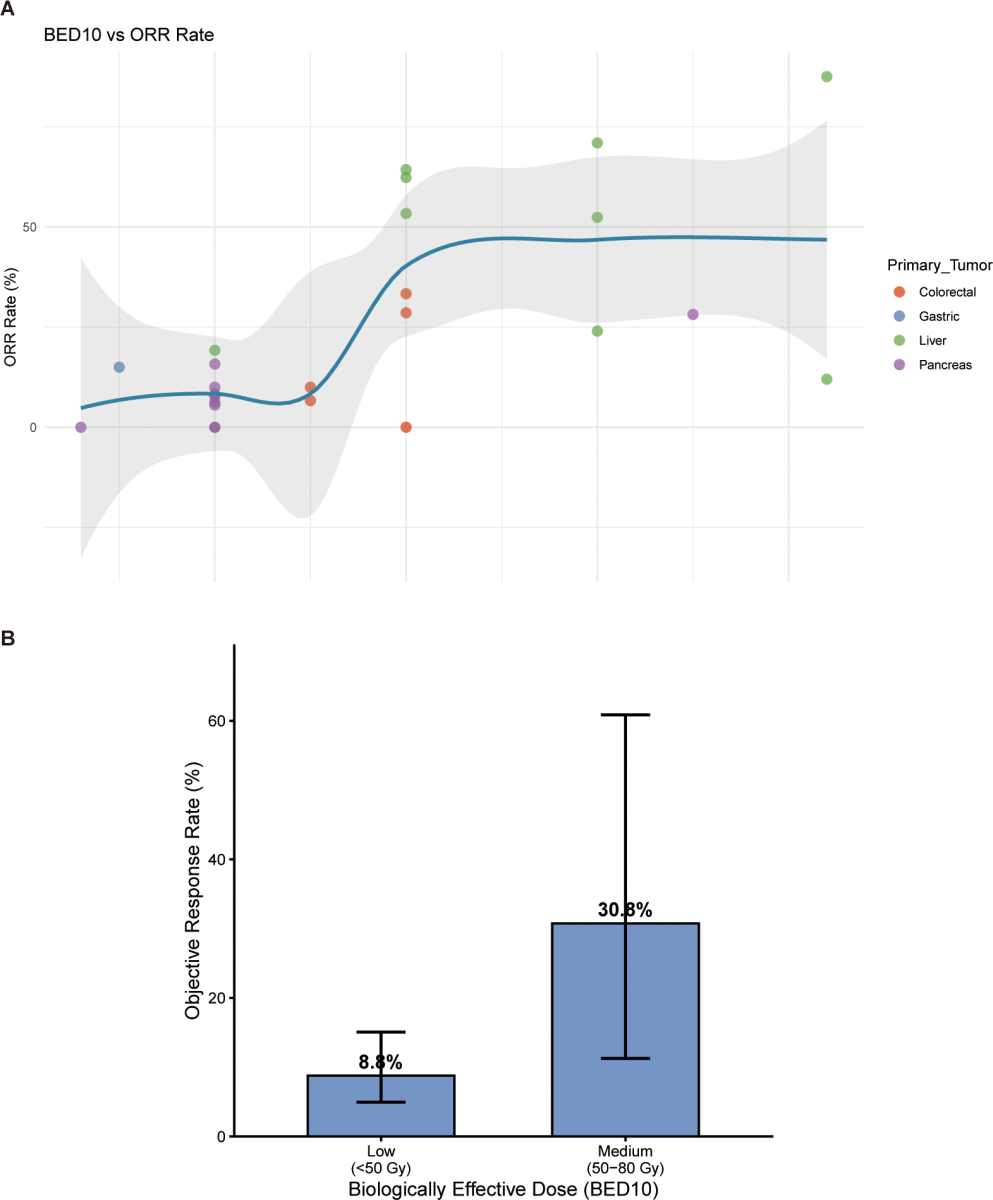
Dose-exploratory analysis. **(A)** Scatter plot showing the relationship between biologically effective dose (BED10) and objective response rate (ORR) across studies (n=23). Each point represents one study, colored by tumor type. The blue shaded area represents the 95% confidence band. **(B)** Bar chart showing pooled ORR rates stratified by BED10 dose category: Low (<50 Gy), Medium (50–80 Gy), and High (>80 Gy). Error bars represent 95% CIs. Higher ablative doses (>80 Gy) were associated with numerically higher response rates.

The abscopal effect was quantitatively assessed in 9 studies, with a mean observed rate of 26.2% (range: 5.6%–61.5%) (**Supplementary Figure 3**). The highest rates were reported in HCC studies (up to 61.5%), suggesting a strong immunogenic potential in this tumor type. Dose-exploratory analysis (**Figure 5**) indicated that the median BED10 utilized was 60 Gy. A scatter plot of BED10 versus ORR suggested a positive correlation, where higher ablative doses appeared associated with improved response rates, particularly in HCC, although this trend requires validation in larger cohorts (**Supplementary Figure 4**).

### 3.6 Publication bias assessment

Assessment of publication bias using Egger’s test revealed no significant small-study effects for ORR (p = 0.096), OS (p = 0.56), or PFS (p = 0.12) (**Supplementary Figure 5**). However, significant bias was detected for DCR (p = 0.002), suggesting that negative results regarding disease stabilization might be underreported.

### 3.7 Sensitivity analyses

To assess the robustness of our findings, leave-one-out sensitivity analyses were performed (**Supplementary Figure 6**). The pooled ORR remained statistically significant across all iterations (range: 3.64–7.48). Remarkably, the pooled HRs for OS (range: 0.38–0.48) and PFS (range: 0.42–0.45) showed minimal variation, confirming the stability of the survival benefit. Furthermore, a sensitivity analysis excluding small studies (n < median sample size) confirmed the ORR benefit (OR = 3.43; 95% CI: 1.25–9.46).

## 4 Discussion

This systematic review and meta-analysis of 25 studies involving 911 patients provides a comprehensive evaluation of SBRT combined with ICIs in advanced gastrointestinal (GI) cancers. The principal findings demonstrate that this combination confers a statistically significant and clinically meaningful improvement across multiple efficacy endpoints. Specifically, the combination yielded a 5.3-fold increase in the odds of objective response and a robust 57% reduction in the risk of both death (HR 0.43) and disease progression (HR 0.43) compared to control therapies. Importantly, these efficacy gains were achieved without a statistically significant increase in high-grade toxicity (RD -0.09). The abscopal effect was observed at a mean rate of 26.2%, suggesting that systemic immune activation is achievable in a subset of patients.

The profound survival benefit (HR 0.43 for both OS and PFS) suggests that SBRT and ICIs effectively alter the natural history of GI malignancies. This synergy aligns with the biological rationale that SBRT-induced immunogenic cell death (ICD) transforms the tumor into an *in situ* vaccine by releasing tumor antigens and DAMPs (11, 13). This process primes cytotoxic T-cells, while ICIs prevent their exhaustion, overcoming the “cold” microenvironment typical of GI tumors.

Our analysis revealed significant tumor-specific heterogeneity. Hepatocellular carcinoma (HCC) was the most responsive (ORR 53.0%), likely due to the liver’s unique tolerogenic environment and chronic inflammation, which may render it more susceptible to radiation-induced immune modulation (33, 34, 41). In contrast, the modest benefit in pancreatic cancer (PDAC) reflects its dense desmoplastic stroma that physically limits T-cell infiltration (22, 35). For metastatic colorectal cancer (mCRC), specifically the MSS/pMMR subset, the lack of significant benefit confirms the challenge of overcoming intrinsic resistance. MSS CRC tumors typically exhibit an ‘immune-excluded’ or ‘immune-desert’ microenvironment characterized by a dense fibrotic stroma and low tumor mutational burden. Although SBRT can induce immunogenic cell death, this effect alone appears insufficient to penetrate the physical stromal barrier or reverse the profound immunosuppressive signaling (e.g., TGF-β pathway) in these ‘cold’ tumors (55, 56).

Compared to early optimistic reports that suggested higher rates of the abscopal effect, our calculated rate of 26.2% provides a more realistic clinical benchmark. While earlier novel monotherapy trials in advanced GI settings often showed only incremental gains, the HR of 0.43 observed here surpasses many current standard-of-care results. Furthermore, the safety profile (Grade ≥3 AEs at 14.6%) is comparable to historical ICI monotherapy rates, confirming that adding ablative radiation does not compromise safety, a finding that corroborates prior Phase I data but extends it to a larger meta-cohort (57).

A major strength of this study is the rigorous exclusion of conventional fractionation (e.g., IMRT) to ensure the homogeneity of the SBRT intervention, addressing a common weakness in previous reviews. However, several limitations remain. First, the majority of included studies were single-arm or retrospective, with only seven controlled trials, introducing potential selection bias. Second, significant clinical heterogeneity existed in SBRT doses and ICI agents. Third, while survival outcomes were robust, significant publication bias was detected for the Disease Control Rate (DCR), suggesting that studies with negative stabilization results might be underreported. Finally, the lack of individual patient data precluded a granular analysis of predictive biomarkers like PD-L1 or TMB.

The potential dose-dependent trend observed between BED10 and abscopal rates suggests a biological threshold for triggering systemic immunity. Clinically, this supports the use of higher ablative doses (e.g., BED10 >60 Gy) in future trials, provided normal tissue constraints are respected (13, 14). However, these findings should be interpreted with caution, as this specific dose-response analysis was based on a limited subset of five studies reporting quantitative abscopal data. Consequently, the statistical power to detect a definitive relationship is limited, and these results should be viewed as hypothesis-generating rather than conclusive.

Consequently, future research should prioritize large-scale randomized controlled trials to confirm the survival benefits identified in this meta-analysis. Efforts should also focus on developing histology-specific strategies, such as combining SBRT with novel immunomodulators or targeted agents in colorectal cancer to overcome desmoplastic or “immune-desert” microenvironments. Additionally, the integration of predictive biomarkers is essential to identify patients most likely to exhibit the abscopal response, thereby optimizing patient selection for this resource-intensive multimodal therapy and ensuring the most effective application of combined SBRT and immune checkpoint inhibition.

## 5 Conclusion

This meta-analysis provides compelling evidence that the combination of SBRT and ICIs is an effective and safe treatment strategy for patients with advanced gastrointestinal cancers, offering significant improvements in response rates, disease control, and survival compared to control therapies. The magnitude of benefit is most substantial in HCC, evident in PDAC, but remains elusive in MSS/pMMR colorectal cancer, highlighting the need for tumor-specific strategy development. Future efforts should focus on conducting large-scale randomized phase III trials to confirm these benefits, optimizing radiation dose-schedules for immune potentiation, defining robust biomarkers for patient selection, and developing even more effective multi-modal regimens for resistant tumor types. The integration of precision radiotherapy with immunotherapy represents a promising frontier in the management of advanced GI malignancies.

## Data Availability

The raw data supporting the conclusions of this article will be made available by the authors, without undue reservation.
