# Supplementary material for: Effectiveness, safety, and the abscopal effect of stereotactic body radiation therapy combined with immune checkpoint inhibitors in advanced gastrointestinal cancers: a systematic review and meta-analysis
Source: Front Oncol. 2026 Mar 10;16:1775732. doi: 10.3389/fonc.2026.1775732 (PMC13008683; doi:10.3389/fonc.2026.1775732)
Supplement: Supplementary file 1 [file DataSheet1.docx]

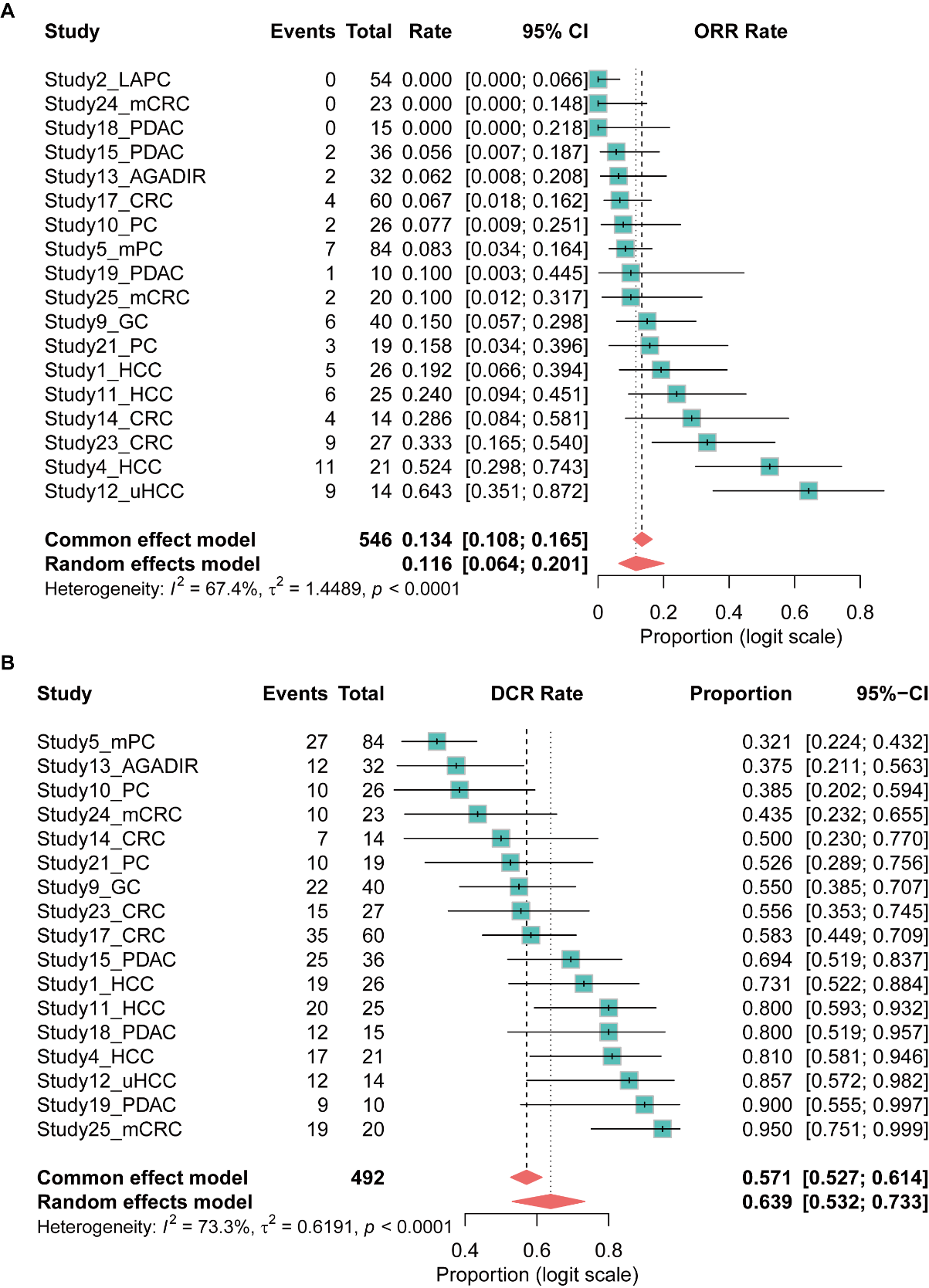


Supplementary Figure S1. Efficacy outcomes in single-arm studies. (A) Forest plot of pooled objective response rate (ORR) from single-arm studies (n=18). (B) Forest plot of pooled disease control rate (DCR) from single-arm studies (n=17). Analyses were performed using a generalized linear mixed model (GLMM) with logit transformation.


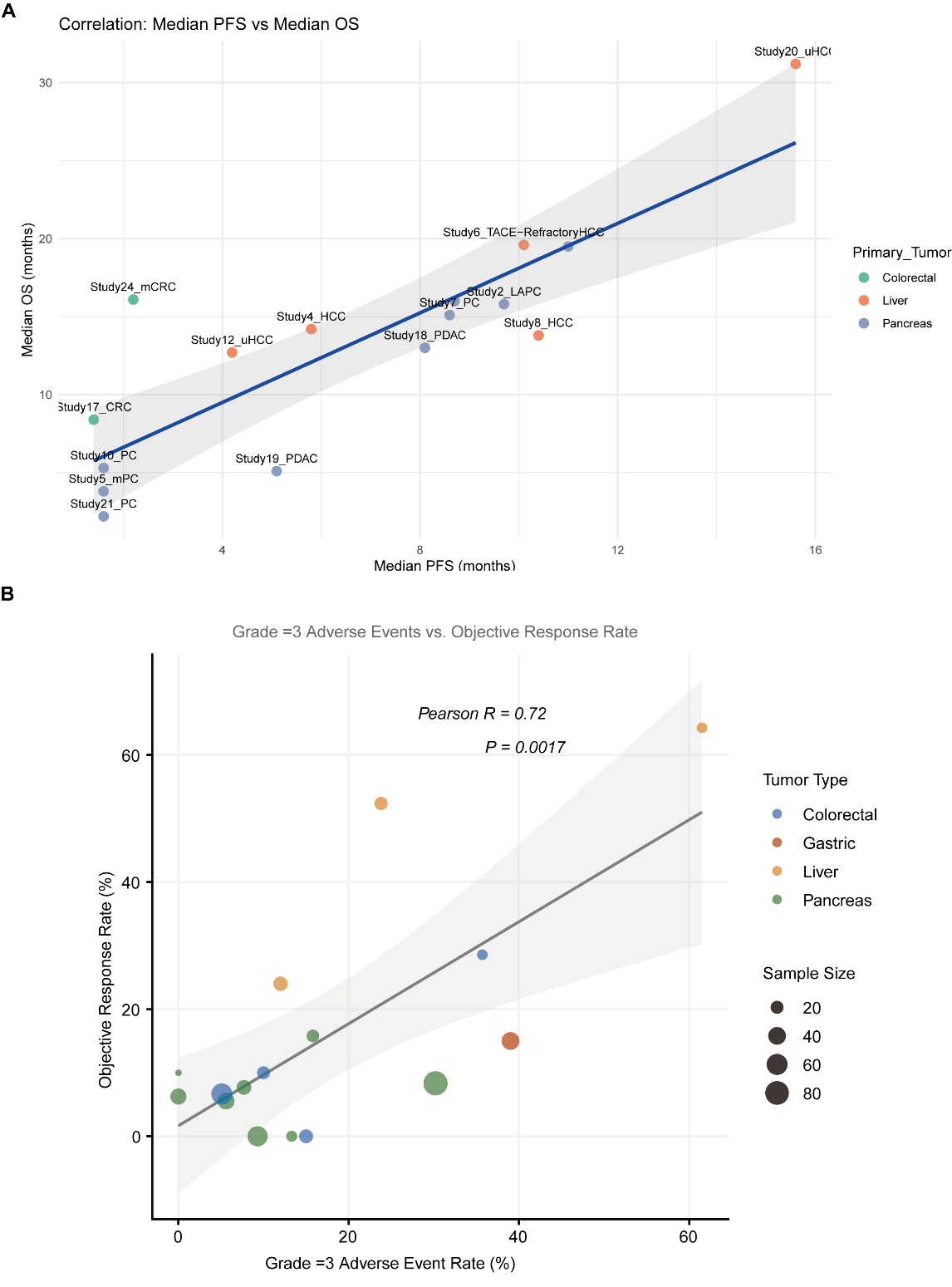


Supplementary Figure S2. Correlation analyses. (A) Scatter plot showing a strong positive correlation (Pearson’s r > 0.8) between median progression-free survival (PFS) and median overall survival (OS). (B) Scatter plot showing the correlation between Grade ≥3 adverse event (AE) rate and objective response rate (ORR). The lack of a strong positive correlation suggests that higher efficacy is not strictly dependent on higher toxicity.


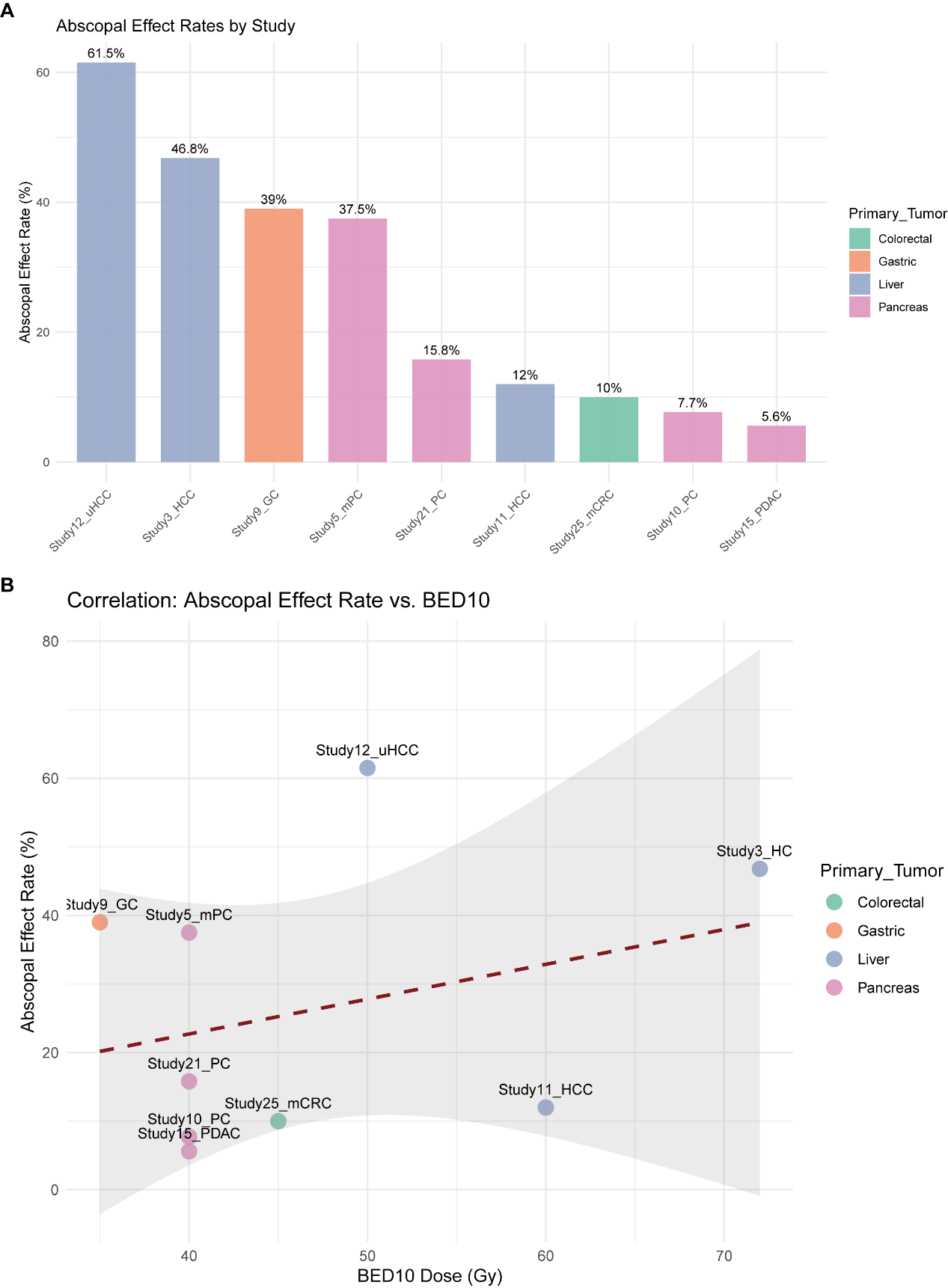


Supplementary Figure S3. Abscopal effect analysis. (A) Bar chart showing the reported rates of the abscopal effect across studies with available quantitative data (n=9). (B) Scatter plot exploring the relationship between SBRT dose (BED10) and the incidence of the abscopal effect.


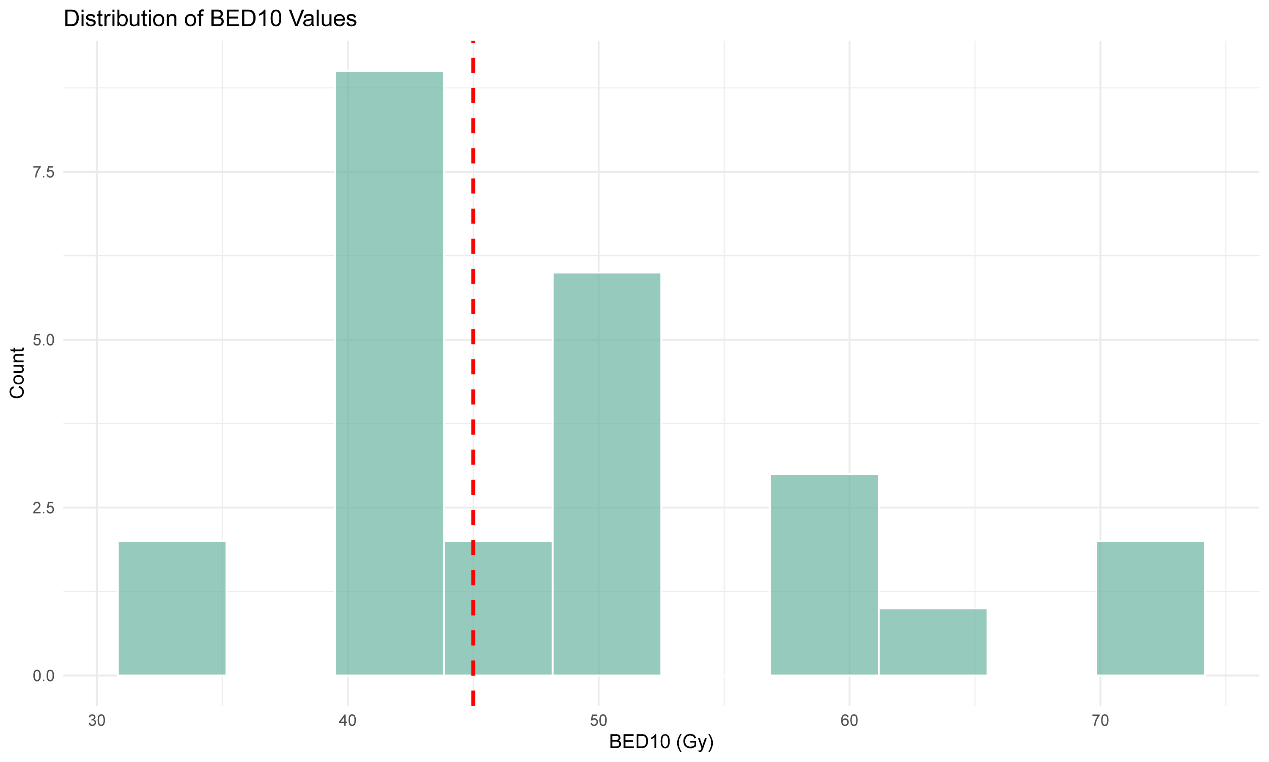


Supplementary Figure S4. Distribution of SBRT doses. Histogram showing the distribution of biologically effective doses (BED10) utilized across all included studies, with a median dose of 60 Gy.


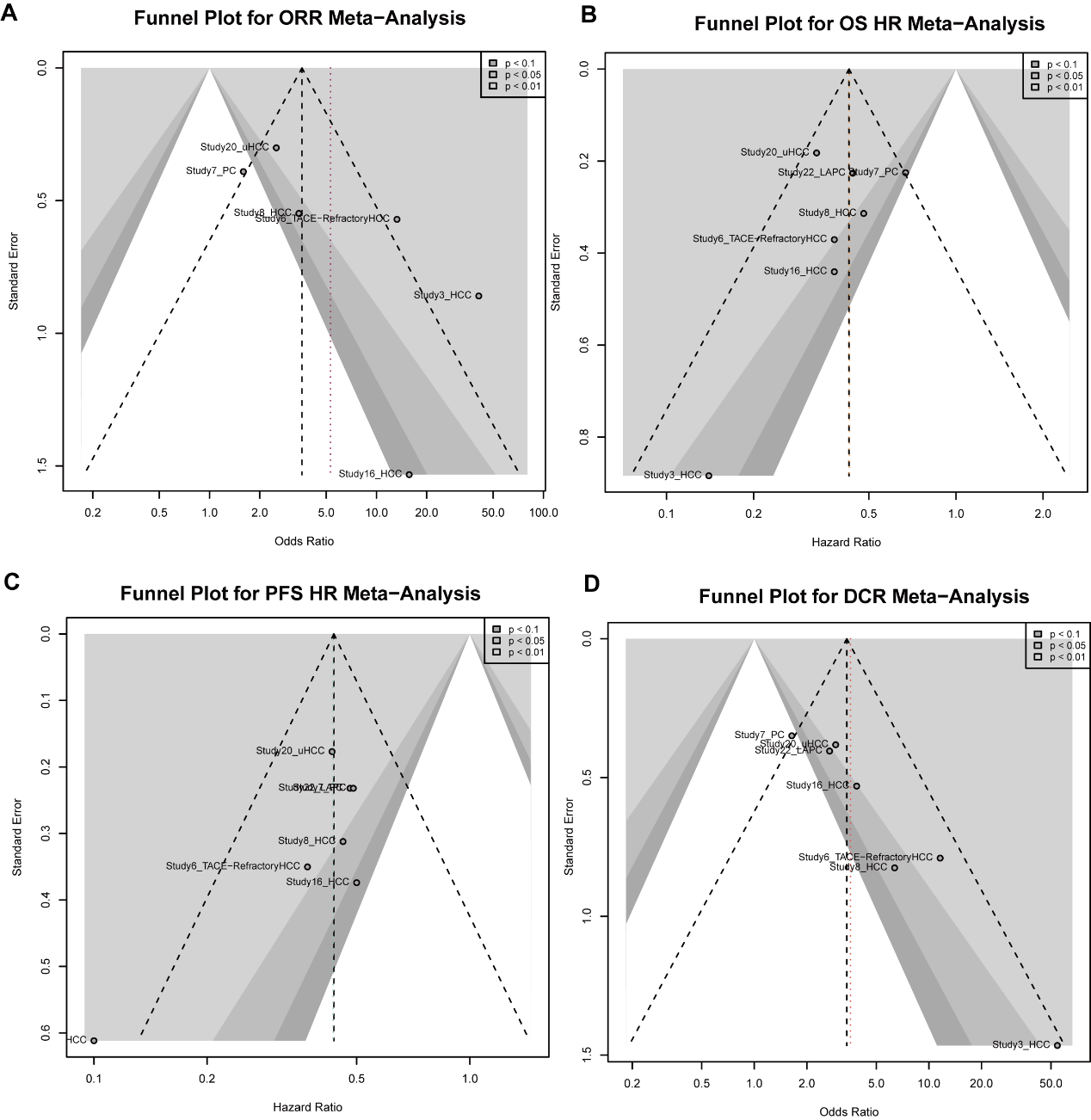


Supplementary Figure S5. Publication bias assessment.

Funnel plots for (A) ORR, (B) OS HR, (C) PFS HR, and (D) DCR. Shaded regions represent significance levels (p<0.10, p<0.05, p<0.01). Egger’s test indicated no significant bias for survival outcomes but potential bias for DCR (p=0.002).


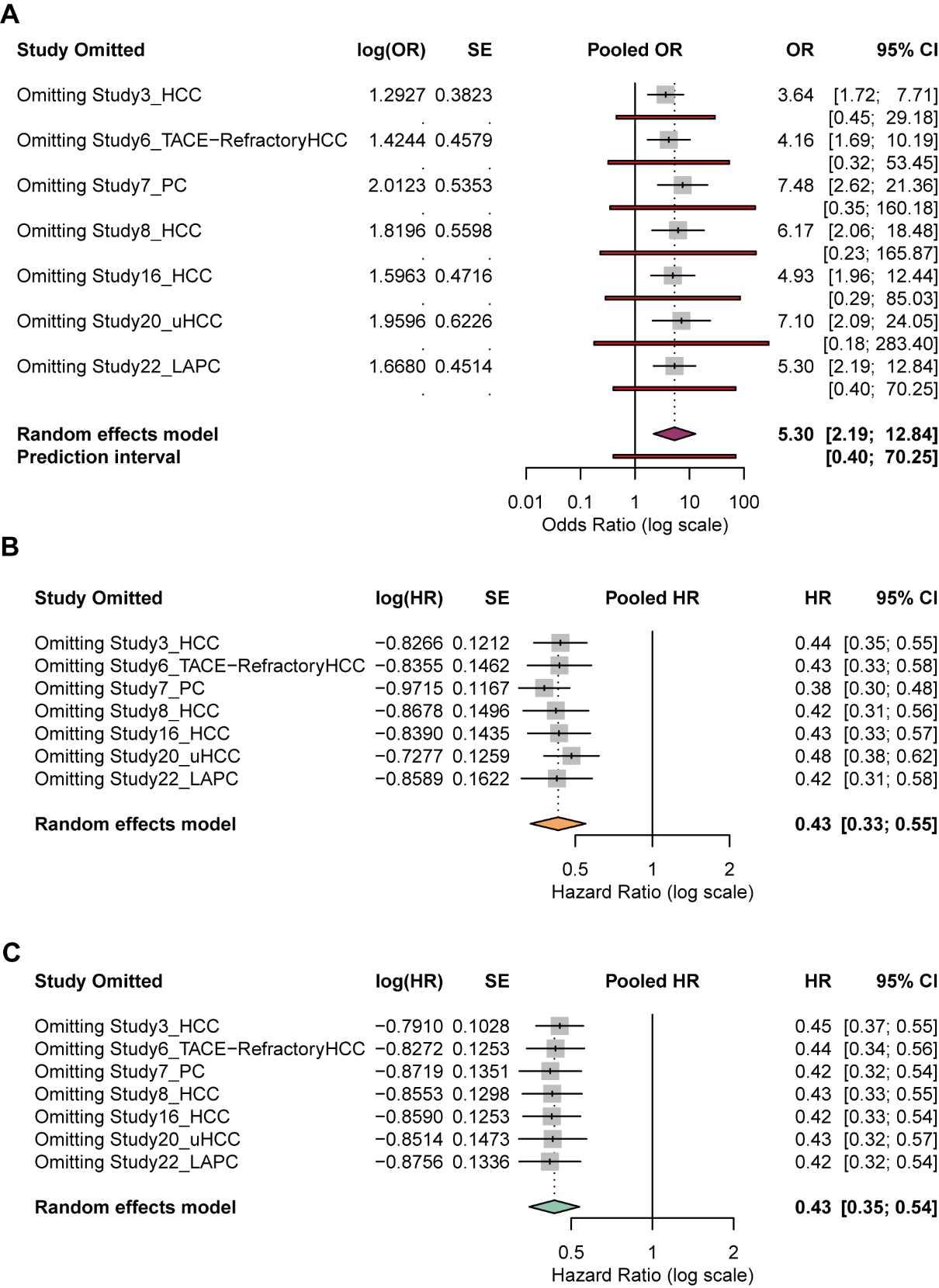


Supplementary Figure S6. Sensitivity analyses. Leave-one-out sensitivity analysis plots for (A) ORR, (B) OS HR, and (C) PFS HR. Each row represents the pooled estimate when the named study is omitted. The results show that the pooled estimates remained stable, confirming the robustness of the primary findings.
