# Supplementary material for: Effectiveness, safety, and the abscopal effect of stereotactic body radiation therapy combined with immune checkpoint inhibitors in advanced gastrointestinal cancers: a systematic review and meta-analysis
Source: Front Oncol. 2026 Mar 10;16:1775732. doi: 10.3389/fonc.2026.1775732 (PMC13008683; doi:10.3389/fonc.2026.1775732)
Supplement: Supplementary file 3 [file DataSheet3.docx]

**ClinicalTrials.gov：333**

﻿Condition: （"Gastrointestinal Neoplasms" OR "Colorectal Cancer" OR "Gastric Cancer" OR "Esophageal Cancer" OR "Pancreatic Cancer" OR "Biliary Tract Cancer" OR "Hepatocellular Carcinoma"）AND（"Advanced Neoplasms" OR "Metastatic Neoplasms" OR "Unresectable Neoplasms"）

﻿Intervention: （"Stereotactic Body Radiation Therapy" OR "SBRT" OR "Stereotactic Ablative Radiotherapy" OR "SABR" OR "Hypofractionated Radiotherapy"）AND（"Immune Checkpoint Inhibitors" OR "PD-1 Inhibitors" OR "PD-L1 Inhibitors" OR "CTLA-4 Inhibitors" OR "Nivolumab" OR "Pembrolizumab" OR "Atezolizumab" OR "Durvalumab" OR "Ipilimumab" OR "Camrelizumab" OR "Sintilimab"）

Study type: completed studies

**Cochrane Library: 294**

#1 MeSH descriptor: [Gastrointestinal Neoplasms] explode all trees 6573

#2 MeSH descriptor: [Colorectal Neoplasms] explode all trees 11738

#3 MeSH descriptor: [Esophageal Neoplasms] explode all trees 3113

#4 MeSH descriptor: [Pancreatic Neoplasms] explode all trees 3566

#5 MeSH descriptor: [Biliary Tract Neoplasms] explode all trees 624

#6 MeSH descriptor: [Hepatocellular Carcinoma] explode all trees 7000

#7 (advanced OR unresectable OR metastatic OR "stage IV" OR oligometastatic OR refractory OR recurrent):ti,ab,kw (Word variations have been searched) 162507

#8 (gastrointestinal cancer* OR gastric cancer* OR colorectal cancer* OR esophageal cancer* OR pancreatic cancer* OR biliary cancer* OR liver cancer* OR cholangiocarcinoma*):ti,ab,kw (Word variations have been searched) 56929

#9 (SBRT OR "stereotactic body radiation therapy" OR SABR OR "stereotactic ablative radiotherapy" OR "hypofractionated radiotherapy" OR "stereotactic radiotherapy"):ti,ab,kw (Word variations have been searched) 2583

#10 MeSH descriptor: [Radiotherapy] explode all trees 45559

#11 MeSH descriptor: [Immune Checkpoint Inhibitors] explode all trees 2039

#12 MeSH descriptor: [Nivolumab] explode all trees 3678

#13 MeSH descriptor: [Pembrolizumab] explode all trees 4177

#14 MeSH descriptor: [Atezolizumab] explode all trees 1809

#15 MeSH descriptor: [Durvalumab] explode all trees 1571

#16 MeSH descriptor: [Ipilimumab] explode all trees 2202

#17 (camrelizumab OR sintilimab OR toripalimab OR tremelimumab OR cemiplimab OR avelumab):ti,ab,kw (Word variations have been searched) 2168

#18 ((PD-1 NEXT inhibitor*) OR (PD-L1 NEXT inhibitor*) OR (CTLA-4 NEXT inhibitor*) OR (immune NEXT checkpoint NEXT blockade*)):ti,ab,kw (Word variations have been searched) 1437

#19 #11 OR #12 OR #13 OR #14 OR #15 OR #16 OR #17 OR #18 13310

#20 #9 OR #10 45843

#21 #1 OR #2 OR #3 OR #4 OR #5 OR #6 OR #8 65278

#22 #21 AND #7 27828

#23 #22 AND #20 AND #19 294

**Pubmed: 2868**

( ("Radiotherapy"[Mesh] OR "Radiotherapy, Intensity-Modulated"[Mesh] OR "SBRT" OR "SABR" OR "stereotactic radiotherapy" OR "stereotactic radiation" OR "stereotactic ablative radiotherapy" OR "hypofractionated radiotherapy" OR "hypofractionated radiation" OR "ablative radiotherapy" OR "radiosurgery" OR "CyberKnife" OR "high-precision radiotherapy" OR "image-guided radiotherapy" OR "IGRT" OR "radiation therapy" OR "radiotherapy") AND ("Immune Checkpoint Inhibitors"[Mesh] OR "Immunotherapy"[Mesh] OR "Programmed Cell Death 1 Receptor"[Mesh] OR "immune checkpoint inhibitor" OR "immune checkpoint blockade" OR "checkpoint inhibition" OR "PD-1" OR "PD-L1" OR "CTLA-4" OR "programmed death 1" OR "programmed death ligand 1" OR "nivolumab" OR "pembrolizumab" OR "atezolizumab" OR "durvalumab" OR "ipilimumab" OR "tremelimumab" OR "camrelizumab" OR "sintilimab" OR "avelumab" OR "cemiplimab" OR "immunotherap*" OR "checkpoint inhibitor" OR "anti-PD-1" OR "anti-PD-L1" OR "anti-CTLA-4" OR "ICI" OR "immune-oncology") ) AND ( ("Gastrointestinal Neoplasms"[Mesh] OR "Stomach Neoplasms"[Mesh] OR "Colorectal Neoplasms"[Mesh] OR "Esophageal Neoplasms"[Mesh] OR "Pancreatic Neoplasms"[Mesh] OR "Biliary Tract Neoplasms"[Mesh] OR "Liver Neoplasms"[Mesh]) OR (gastrointestinal cancer OR gastric cancer OR stomach cancer OR colorectal cancer OR colon cancer OR rectal cancer OR esophageal cancer OR oesophageal cancer OR pancreatic cancer OR biliary cancer OR cholangiocarcinoma OR "hepatocellular carcinoma" OR HCC OR liver cancer OR "GI cancer" OR "digestive system cancer" OR "digestive tract cancer" OR "gastrointestinal tumor" OR "gastric tumor" OR "colorectal tumor" OR "esophageal tumor" OR "pancreatic tumor" OR "hepatobiliary cancer") ) NOT ("Case Reports"[Publication Type] OR "Review"[Publication Type])

**Web of Science Core Collection: 113**

("Stereotactic Body Radiation Therapy" OR "SBRT" OR "Stereotactic Radiotherapy" OR "Stereotactic Ablative Radiotherapy" OR "SABR" OR "Hypofractionated Radiotherapy" OR "Stereotactic Radiation Therapy")) (Topic) and (("Immune Checkpoint Inhibitor*" OR "PD-1 Inhibitor*" OR "PD-L1 Inhibitor*" OR "CTLA-4 Inhibitor*" OR "Immune Checkpoint Blockade" OR "Checkpoint Inhibitor*") OR nivolumab OR pembrolizumab OR atezolizumab OR durvalumab OR ipilimumab OR camrelizumab OR sintilimab) (All Fields) and (("Gastrointestinal Neoplasm*" OR "Stomach Cancer*" OR "Colorectal Cancer*" OR "Esophageal Cancer*" OR "Pancreatic Cancer*" OR "Biliary Tract Cancer*" OR "Hepatocellular Carcinoma*" OR "Cholangiocarcinoma*") OR ("Gastrointestinal Tumor*" OR "Digestive System Cancer*")) (All Fields)

**Embase: 250**

1 'gastrointestinal neoplasm'/de OR 'colorectal cancer'/de OR 'gastric cancer'/de OR 'esophageal cancer'/de OR 'pancreatic cancer'/de OR 'biliary tract cancer'/de OR 'hepatocellular carcinoma'/de OR 'cholangiocarcinoma'/de

2 'gastrointestinal neoplasm'/exp OR 'colorectal cancer'/exp OR 'advanced cancer'/exp OR 'metastatic neoplasm'/exp OR 'unresectable tumor'/exp OR 'oligometastatic neoplasm'/exp

3 ("colorectal cancer*":ti,ab,kw OR "gastric cancer*":ti,ab,kw OR "esophageal cancer*":ti,ab,kw OR "pancreatic cancer*":ti,ab,kw OR "biliary tract cancer*":ti,ab,kw OR "hepatocellular carcinoma*":ti,ab,kw OR cholangiocarcinoma:ti,ab,kw OR "gastrointestinal tumor*":ti,ab,kw OR "digestive system cancer*":ti,ab,kw)

4 (advanced:ti,ab,kw OR metastatic:ti,ab,kw OR unresectable:ti,ab,kw OR "stage IV":ti,ab,kw OR "stage 4":ti,ab,kw OR oligometastatic:ti,ab,kw)

5 #1 AND (#2 OR #3 OR #4)

6 'stereotactic body radiation therapy'/de OR 'stereotactic ablative radiotherapy'/de OR 'stereotactic radiotherapy'/de OR 'hypofractionated radiotherapy'/de

7 SBRT:ti,ab,kw OR SABR:ti,ab,kw OR "stereotactic body radiation therapy":ti,ab,kw OR "stereotactic ablative radiotherapy":ti,ab,kw OR "hypofractionated radiotherapy":ti,ab,kw OR "stereotactic radiation":ti,ab,kw

8 #6 OR #7

9 'immune checkpoint inhibitor'/de OR 'pd-1 inhibitor'/de OR 'pd-l1 inhibitor'/de OR 'ctla-4 inhibitor'/de

10 'immune checkpoint inhibitor'/exp OR 'immune checkpoint blockade'/exp

11 (nivolumab:ti,ab,kw OR pembrolizumab:ti,ab,kw OR atezolizumab:ti,ab,kw OR durvalumab:ti,ab,kw OR ipilimumab:ti,ab,kw OR camrelizumab:ti,ab,kw OR sintilimab:ti,ab,kw OR tremelimumab:ti,ab,kw OR toripalimab:ti,ab,kw OR cemiplimab:ti,ab,kw)

12 ("PD-1 inhibitor*":ti,ab,kw OR "PD-L1 inhibitor*":ti,ab,kw OR "CTLA-4 inhibitor*":ti,ab,kw OR "immune checkpoint":ti,ab,kw OR "checkpoint blockade":ti,ab,kw)

13 #9 OR #10 OR #11 OR #12

14 'randomized controlled trial'/de OR 'controlled clinical trial'/de OR 'double blind procedure'/de OR 'single blind procedure'/de OR 'prospective study'/de OR 'clinical trial'/de OR 'retrospective study'/de OR 'cohort study'/de OR 'retrospective cohort study'/de

15 ("randomized controlled trial":ti,ab,kw OR RCT:ti,ab,kw OR (randomi?ed NEAR/2 controlled):ti,ab,kw OR (double NEAR/1 blind*):ti,ab,kw OR (single NEAR/1 blind*):ti,ab,kw OR "placebo controlled":ti,ab,kw OR "prospective study":ti,ab,kw OR "clinical trial":ti,ab,kw OR "retrospective study":ti,ab,kw OR "cohort study":ti,ab,kw OR "retrospective cohort study":ti,ab,kw OR "historical cohort":ti,ab,kw)

16 #14 OR #15

17 #5 AND #8 AND #13 AND #16
